# Genome-Wide Association Study of the Genetic Basis of Effective Tiller Number in Rice

**DOI:** 10.1186/s12284-021-00495-8

**Published:** 2021-06-25

**Authors:** Mengmeng Ren, Minghan Huang, Haiyang Qiu, Yan Chun, Lu Li, Ashmit Kumar, Jingjing Fang, Jinfeng Zhao, Hang He, Xueyong Li

**Affiliations:** 1 National Key Facility for Crop Gene Resources and Genetic Improvement, Institute of Crop Sciences, Chinese Academy of Agricultural Sciences, Beijing, 100081 China; 2 School of Advanced Agriculture Sciences and School of Life Sciences, State Key Laboratory of Protein and Plant Gene Research, Peking-Tsinghua Center for Life Sciences, Peking University, Beijing, 100871 China; 3 Peking University Institute of Advanced Agricultural Sciences, Weifang, 261325 Shandong China

**Keywords:** Effective tiller number, GWAS, Rice

## Abstract

**Background:**

Effective tiller number (ETN) has a pivotal role in determination of rice (*Oryza sativa* L*.*) grain yield. ETN is a complex quantitative trait regulated by both genetic and environmental factors. Despite multiple tillering-related genes have been cloned previously, few of them have been utilized in practical breeding programs.

**Results:**

In this study, we conducted a genome-wide association study (GWAS) for ETN using a panel of 490 rice accessions derived from the 3 K rice genomes project. Thirty eight ETN-associated QTLs were identified, interestingly, four of which colocalized with the *OsAAP1*, *DWL2*, *NAL1*, and *OsWRKY74* gene previously reported to be involved in rice tillering regulation. Haplotype (Hap) analysis revealed that Hap5 of *OsAAP1*, Hap3 and 6 of *DWL2*, Hap2 of *NAL1*, and Hap3 and 4 of *OsWRKY74* are favorable alleles for ETN. Pyramiding favorable alleles of all these four genes had more enhancement in ETN than accessions harboring the favorable allele of only one gene. Moreover, we identified 25 novel candidate genes which might also affect ETN, and the positive association between expression levels of the *OsPILS6b* gene and ETN was validated by RT-qPCR. Furthermore, transcriptome analysis on data released on public database revealed that most ETN-associated genes showed a relatively high expression from 21 days after transplanting (DAT) to 49 DAT and decreased since then. This unique expression pattern of ETN-associated genes may contribute to the transition from vegetative to reproductive growth of tillers.

**Conclusions:**

Our results revealed that GWAS is a feasible way to mine ETN-associated genes. The candidate genes and favorable alleles identified in this study have the potential application value in rice molecular breeding for high ETN and grain yield.

**Supplementary Information:**

The online version contains supplementary material available at 10.1186/s12284-021-00495-8.

## Background

Rice (*Oryza sativa* L.) is one of the main staple crops worldwide and improving rice yield is an urgent need for the increasing world’s population (Lobell et al. 2011). Effective tiller number (ETN) per plant is an essential yield component for rice and affected by both genetic and environmental factors (Xing & Zhang 2010). Rice tiller derives from two processes, axillary meristem (AM) formation and tiller bud outgrowth (Wang & Li 2011), which are regulated by many genes. *MOC1,* encoding a GRAS domain transcription factor, plays a pivotal role in AM formation, and the loss-of-function *moc1* mutant shows a monoculm phenotype (Li et al. 2003). *MOC3*/*OsWUS*/*TILLERS ABSENT1* (*TAB1*) is also indispensable for tiller bud formation (Lu et al. 2015b), which may promote AM initiation by inducing the expression of *OSH1* (Tanaka et al. 2015). Besides, LAX PANICLE1 (LAX1) and LAX2 can physically interact with MOC1 and also regulate AM formation (Oikawa & Kyozuka 2009; Tabuchi et al. 2011). Tiller bud outgrowth is suppressed by a newly discovered plant hormone, strigolactones (SLs) (Gomez-Roldan et al. 2008). Typical SL mutants display dwarf and increased tillering, such as *d27* (Lin et al. 2009), *htd1*/*d17* (Zou et al. 2006), and *d10* (Arite et al. 2007; Yuan et al. 2013), whose responsible genes are involved in SL synthesis; *htd2*/*d14* (Liu et al. 2009), *d3* (Yasuno et al. 2009; Zhao et al. 2014) and *d53* (Zhou et al. 2013; Jiang et al. 2013), whose responsible genes are involved in SL signaling.

Besides SL, other plant hormones can also affect rice tiller growth. For instance, overexpression of *OsPIN2* or *OsPIN9,* both encoding an auxin efflux transporter, led to increased tiller number (TN) (Chen et al. 2012; Hou et al. 2021). *OsCKX9,* encoding a cytokinin (CK) catabolic enzyme, suppressed tillering upon transcriptional activation by SL (Duan et al. 2019). DELLA protein SLENDER RICE 1, a repressor of gibberellin (GA) signaling, inhibits MOC1 degradation to regulate both tillering and plant height (Liao et al. 2019). Tiller growth could also be influenced by some genes independent of plant hormones. For example, *MOC2* encodes a fructose-1,6-bisphosphatase participating in sucrose synthesis, and the *moc2* mutant also shows monoculm phenotype (Koumoto et al. 2013). Hd3a is the homolog of Arabidopsis FLOWERING LOCUST (FT) protein in rice, which is transported from the phloem to shoot apical cells and promotes lateral branching (Zhao et al. 2015). The *rcn1* mutant displays monoculm phenotype and the responsible gene *OsABCG5* encodes an ATP-binding cassette protein required for rice shoot branching (Yasuno et al. 2009).

Fast-growing next-generation sequencing (NGS) technology has become a cheaper solution for genotyping, which makes it possible to use high-throughput single nucleotide polymorphism (SNP) markers to perform GWAS (Liu & Yan 2019). Many genes controlling important agronomic traits have been identified using this method in recent years, such as *OsSPL13* controlling rice grain size (Si et al. 2016), *OsNPF6.1* associated with nitrogen use efficiency in rice (Tang et al. 2019), *ZmVPP1* contributing to drought tolerance in maize (Wang et al. 2016), and *ZmFBL41* conferring banded leaf and sheath blight resistance in maize (Li et al. 2019). As an essential yield-related trait, rice TN has also been studied by GWAS. A previous study on 14 agronomic traits had identified eight loci associated with TN, distributing on chromosome 1, 2, 4 and 10 (Huang et al. 2010). In another study on 15 agronomic traits using a high-throughput phenotyping facility, an F-box gene *OsFBL20* controlling TN was identified (Yang et al. 2014). A recent GWAS identified 15 novel loci associated with TN variations, and five candidate genes were validated (Jiang et al. 2019). Another study based on TN data in different stages revealed that dynamic change in TN played a key role in determination of panicle number and identified a new gene *OsSAUR27* associated with TN (Ma et al. 2020).

Most genes regulating rice tillering have been cloned from high- or low-tillering mutants which are rarely used as rice breeding materials. However, ETN is a complex trait with a relatively low heritability and contributed by multiple QTLs (Liu et al. 2010). Therefore, in order to clone rice tillering genes potentially used by breeders, more QTLs need to be identified from the natural rice population. In this study, we performed GWAS based on ETN data of a panel of 490 rice accessions grown in two locations, and the objectives are as follows: (1) feasibility analysis of mining ETN genes by GWAS; (2) identify novel genes affecting ETN; (3) probe favorable alleles of ETN-associated genes.

## Methods

### Plant Materials

The 490 rice accessions used in this study were derived from the 3000 Rice Genomes Project (Wang et al. 2018). Information of these rice accessions was shown in Additional file 1: Table S1.

### Phenotyping Analysis

For the field experiments, rice seeds were submerged in clean water at 37 °C for 48 h and transferred to nursery bed for germination. One-month-old seedlings were then transplanted into the paddy field at density of 16 cm × 20 cm and one plant per hill. A completely randomized block design with three replicates was performed. Six plants in the middle of every row were selected for evaluation of ETN with effective tiller being defined as the one bearing more than 10 seeds at maturity stage. The mean value of three replicates was used for analysis. The field experiment was performed in two different years and locations: (1) June to November 2017, Fengcheng city, Jiangxi (JX) Province, China (28°15′N, 115°77′E); (2) December 2017 to April 2018, Sanya city, Hainan (HN) Province, China (18°25′N, 109°51′E). Broad-sense heritability (*H*^*2*^) was calculated based on the following formular: *H* ^*2*^ = *V*_*G*_/(*V*_*G*_ + *V*_*E*_) , where *V*_*G*_ and *V*_*E*_ are genetic and environmental variances.

### Genotyping Data and SNP Filtering

The raw genotype data of the 490 accessions were obtained from the Rice SNP-Seek Database (https://snp-seek.irri.org/) (Alexandrov et al. 2015). A total of 5,877,569, 4,542,091 and 4,995,512 cleaned SNPs were called in the whole, *indica* and *non-indica* (*nonind*) population, respectively. SNPs were filtered utilizing the software PLINK (Purcell et al. 2007) with missing rate < 40% and minor allele frequency (MAF) > 0.05.

### Population Structure and Phylogenetic Analysis

The software ADMIXTURE 1.3 (Alexander et al. 2009) was used to calculate the population structure. A total of 394,572 SNPs pruned by PLINK with *r*^*2*^ > 0.3 were used for the analysis. The ancestral population number (*K*) was assumed ranging from 2 to 6. Principal component analysis (PCA) was performed using PLINK and plotted with R ‘ggplot2’ package (Wickham. 2016). The FastTree software (Price et al. 2009) was used to construct the phylogenetic tree based on the approximately maximum-likelihood method, and the generated Newick file was then visualized on the iTOL website (https://itol.embl.de/) (Letunic & Bork. 2019).

### GWAS and Linkage Disequilibrium (LD) Analysis

GWAS was performed with EMMAX program (Kang et al. 2010). Briefly, we first used PLINK to get the genotype file, and the ETN phenotype data were collected from two locations, Jiangxi and Hainan. The genotype and phenotype file, as well as the Balding-Nichols kinship matrix were used to fit the mixed linear model in EMMAX. The Manhattan and quantile-quantile (Q-Q) plots were generated using the R package “qqman” (Turner. 2018). The pairwise linkage disequilibrium (LD) (*r*^*2*^) was calculated using PopLDdecay software (Zhang et al. 2019). The Pairwise *r*^*2*^ was calculated for the SNPs in 500 kb and averaged in 1 kb across the whole genome. The LD decay rate was measured as the chromosomal distance at which the average pairwise correlation coefficient (*r*^*2*^) decreased to half of its maximum value (Huang et al. 2010). The regional Manhattan plot and LD heatmap were obtained using LDBlockShow software (Dong et al. 2020).

### Haplotype Analysis

The haplotypes of candidate genes were classified using SNPs and InDels (insertion and deletion) in the promoter (2 kb upstream of ATG) and untranslated region (UTR), as well as non-synonymous SNPs in the coding sequence (CDS). Haplotypes with more than 10 accessions were retained to perform one-way variance analysis (ANOVA) in R. Multiple comparisons were conducted using R package “agricolae”.

### Resequencing of *OsPILS6b*

Genomic DNA of 28 accessions (Additional file 1: Table S1) was extracted using standard CTAB method (Doyle 1987), and the 5.9 kb full-length sequence for the *OsPILS6b* gene (including 2.5 kb upstream of ATG) was amplified with high-fidelity DNA polymerase KOD FX (TOYOBO, KFX-101) using the following primers, PILS6bF: CAGTCAAGCATCTCACCCTTT, PILS6bR: AGCCGATTGGTTTAT ACTGGA. PCR product was sequenced directly by the Beijing Genomics Institute. Multiple sequence alignment was performed using MEGA X (Sudhir et al. 2018).

### Reverse Transcription Quantitative-PCR Analysis

Eight, twelve, and eight accessions representing three different haplotypes of *OsPILS6b* were selected from the 490 rice panel (Additional file 1: Table S1), respectively. Shoot bases (about 0.5 cm) of 10-day-old rice seedlings were used for total RNA isolation using TRIzol reagent (Invitrogen). One μg of RNA was treated with DNase I (TAKARA) and then used for cDNA synthesis (TAKARA) according to the manufacturer’s instructions. The rice *OsActin1* gene (*LOC_Os03g50885*) was used as an internal control. The reverse transcription quantitative-PCR (RT-qPCR) primers used for *OsActin1* and *OsPILS6b* were as follows: F1: TCCATCTTGGCATCTCTCAG, R1: GGTACCCTCATCAGGCATCT; F2: ACCTTTGACAGCTGCGATGA, R2: ATAGCAGGGGCTCTTCCTCA. The qRT-PCR was performed on an ABI Prism 7500 instrument (Applied Biosystems) and relative gene expression level was calculated using the 2^-ΔΔCT^ method (Livak & Schmittgen 2001).

## Results

### Population Structure and ETN Variation of the 490 Rice Accessions

**Fig. 1:**
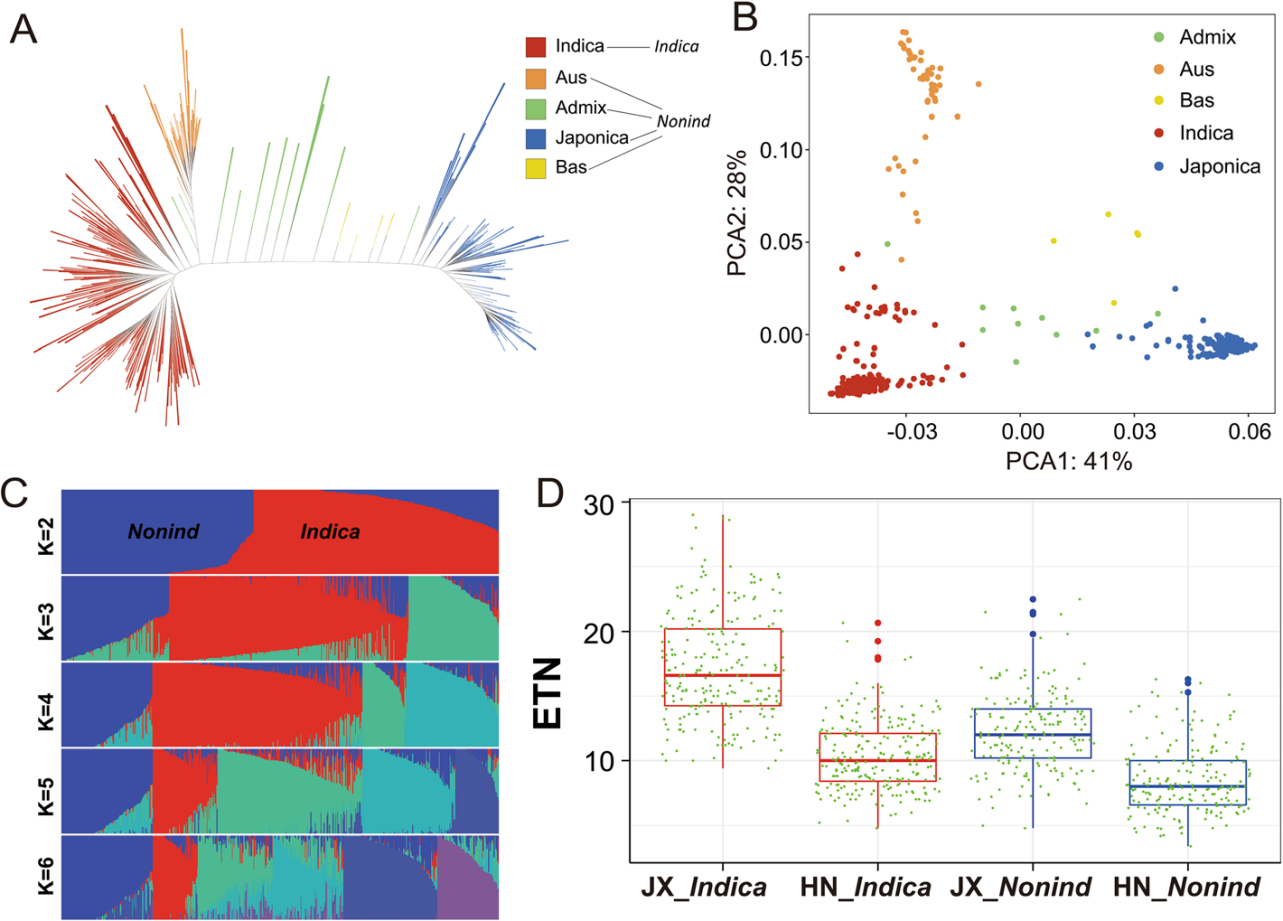
Population structure and ETN variation of 490 rice accessions. (**a**) Phylogenetic tree constructed using LD-pruned SNPs. (**b**) PCA plots for the first two components of 490 rice accessions. (**c**) Population structure determined by ADMIXTURE. Ancestral population number (*K*) ranged from two to six. (**d**) ETN variation of two subpopulations in two locations. Boxplot described ETN distribution of one subpopulation in one location, and each dot around the boxplot represent ETN value of one accession. ETN, effective tiller number; JX, Jiangxi province; HN, Hainan province. Different letters denote significant differences (*P* < 0.05) based on Duncan’s multiple-range test

The 490 rice accessions consist of 235 *Indica*, 194 *Japonica*, 45 *Aus*, 11 *Admixture* (*Admix*) and 5 *Basmati* (*Bas*), according to the K9_group_Admixture (Wang et al. 2018) (Additional file 1: Table S1). Due to the smaller population size compared with that of *Indica*, for the follow-up analysis, *Japonica*, *Aus*, *Admix* and *Bas* were combined into one group named as *non-indica* (*nonind*). Phylogenic tree (Fig. 1a) and population structure analysis (Fig. 1c, Additional file 5: Fig. S1a) demonstrate that these two subpopulations were distinguishable according to their genotype, which was also supported by result of PCA analysis (Fig. 1b). Linkage disequilibrium (LD) analysis showed the LD decay rate was higher in *indica* than *nonind* (Additional file 5: Fig. S1b), consistent with previous studies (Huang et al. 2010; Wang et al. 2018).

This panel of 490 rice accessions was grown in summer 2017 in Jiangxi Province (JX) and winter 2017 in Hainan Province (HN), respectively. ETN variation was quite abundant in our population, ranging from 4.8 to 30 and 3.4 to 20.7 in JX and HN, respectively. Overall, *indica* accessions showed more ETN and wider ETN range than *nonind* accessions, similar to the trend observed in a previous study (Ma et al. 2020). The results also showed that both *indica* and *nonind* accessions had more tillers in JX than in HN (Fig. 1d). We speculated that the short daylight condition in winter in HN promotes flowering and restrains the vegetative growth and tillering in rice. Despite the dramatic environmental difference, ETN in these two locations were slightly correlated, with a correlation coefficient of *R*^*2*^ = 0.45. Broad-sense heritability (*H*^*2*^) of ETN was 0.58, consistent with a previous study (Liu et al. 2010). The relatively low heritability indicates that besides genetic factors, environmental factors also play a critical role in ETN variation.

### Overview of QTLs Associated with ETN Detected by GWAS

**Fig. 2:**
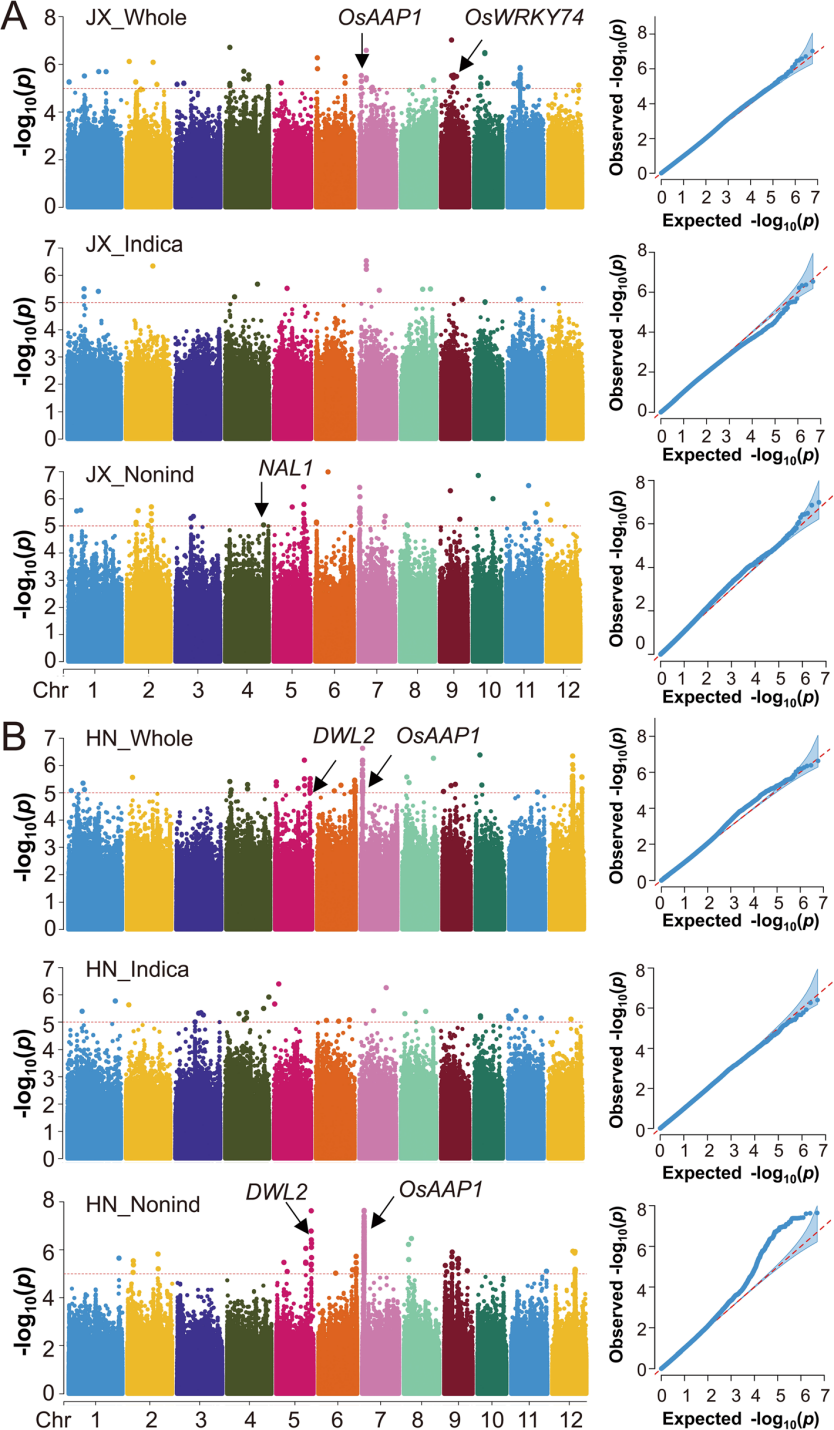
Manhattan plots and quantile-quantile (Q-Q) plots of six GWAS assays. (**a**) GWAS assays for the whole, *indica* and *nonind* population using ETN data in Jiangxi, (**b**) GWAS assays for whole, *indica* and *nonind* population using ETN data in Hainan. Some candidate genes are indicated using arrow heads

As mentioned above, both the population and the location have significant effect on ETN. Therefore, to exclude effect of population structure and environments, we performed six GWAS assays depending on the location and subpopulation, i.e., the whole, *indica* and *nonind* populations in JX and HN, respectively. As reported previously, a QTL was called when there were at least two significant SNPs (*P* ≤ 10E-5) within 200-kb range (Jiang et al. 2019), and the region of a specific QTL was defined as 300 kb flanking the lead SNP (SNP with the lowest *P* value in a cluster) (Guo et al. 2020). By this standard, a total of 38 ETN-associated QTLs were identified (Fig. 2), among which 7 QTLs were detected by two or three GWAS assays. Detailed information of these QTLs was listed in Additional file 2: Table S2. A total of 1134 genes are located in these regions.

### Colocalization of ETN-Associated QTLs with Previously Reported Rice Tillering Genes

**Fig. 3:**
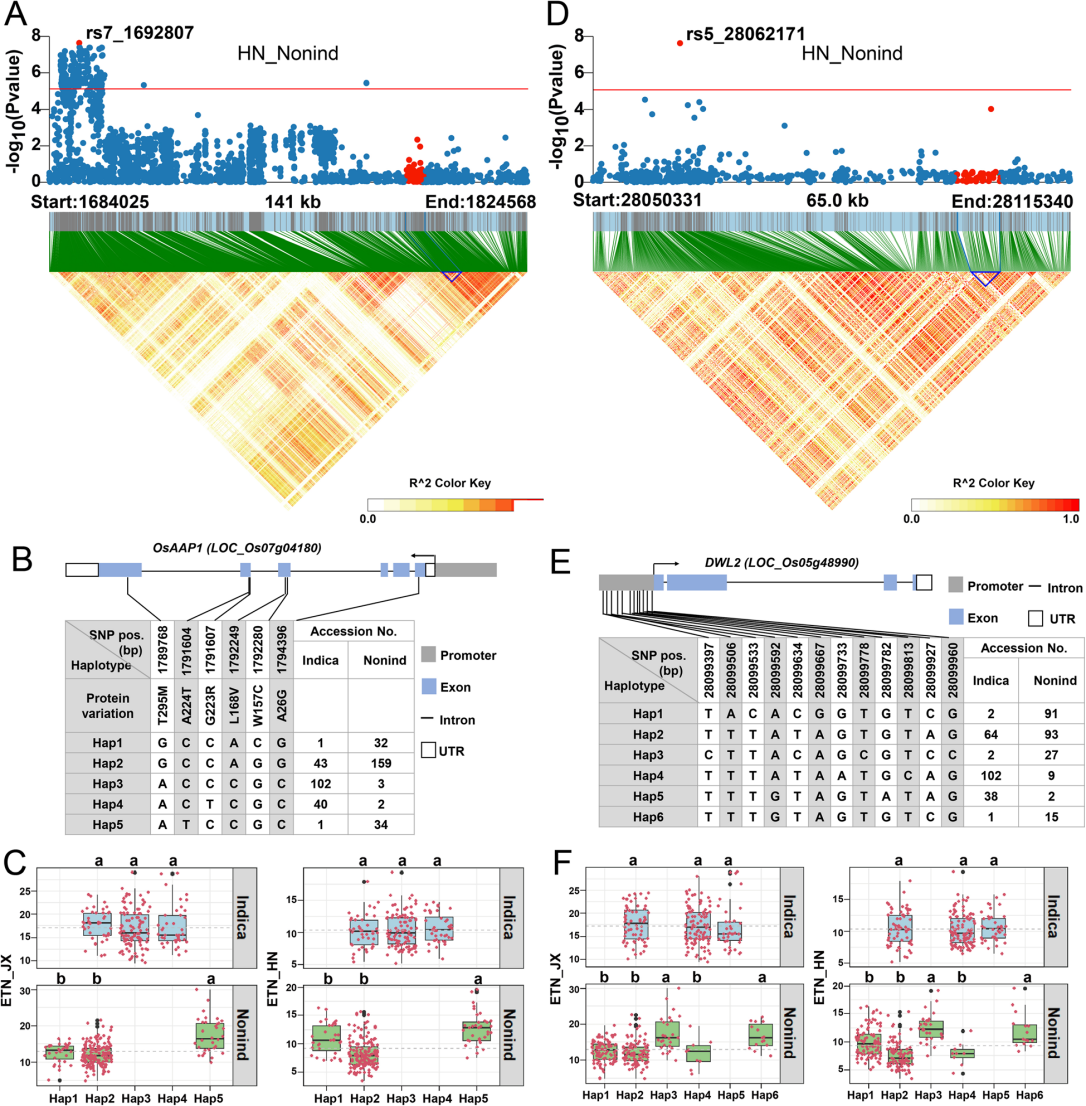
Associated region and haplotype analysis of *OsAAP1* and *DWL2*. (**a, d**) Regional Manhattan plots and LD heatmap of *OsAAP1* (**a**) and *DWL2* (**d**). The single red dot above the red line denotes the lead SNP, the red dots under the red line and the blue triangle in the LD heatmap denote the genomic region of candidate genes. (**b, e**) Gene structure and marker information of *OsAAP1* (**b**) and *DWL2* (**e**)*.* Arrow heads indicate the direction of gene transcription. (**c, f**) Haplotype analysis of *OsAAP1* (**c**) and *DWL2* (**f**). Boxplot described ETN distribution of one subpopulation with one haplotype, and each red dot around the boxplot represents ETN value of one accession. The gray dotted-lines denote average ETN of one subgroup. Different letters denote significant differences (*P* < 0.05) based on Duncan’s multiple-range test

To evaluate the reliability of our GWAS results, we examined whether the detected ETN-associated QTLs could colocalize with some known genes involved in rice tillering. First, we targeted ETN7–3, which was detected in three GWAS assays (JX_whole, HN_whole, and HN_nonind) and showed the most remarkable signal (Fig. 2). Fortunately, the *OsAAP1* gene encoding an amino acid transporter which has recently been reported to affect TN, is located in this region. Overexpression lines of *OsAAP1* showed enhanced TN, whereas RNAi lines showed opposite phenotypes (Ji et al. 2020). *OsAAP1* was located around 102 kb downstream of the peak SNP (rs7_1692807) of ETN7–3. Therefore, we called all the SNPs within 141-kb region flanking *OsAAP1* and investigated the linkage disequilibrium (LD) of *OsAAP1* in the 490 rice accessions panel. The LD heatmap showed that *OsAAP1* is located in a region with relatively high LD (Fig. 3a). Next, we analyzed the haplotype of *OsAAP1* in both *indica* and *nonind* subpopulations. In total, we identified six non-synonymous SNPs in the coding sequence (CDS) (Fig. 3b). Based on these variants, we identified 5 haplotypes of *OsAAP1*. The *indica* and *nonind* subgroup have three different major haplotypes, respectively, and Hap2 is present as a major haplotype in both subgroup. In agreement with the fact that *OsAAP1* was detected in *nonind* rather than *indica* subgroup, there seems no much difference in ETN among the three major haplotypes (Hap2, 3, 4) in *indica* subgroup. However, the ETN varies significantly among the three major haplotypes (Hap1, 2 and 5) in *nonind* subgroup, with Hap5 showing the highest ETN in both JX and HN (Fig. 3c). Therefore, Hap5 is a favorable allele for ETN and introducing this Hap5 allele of *OsAAP1* into other cultivars may contribute to an increase of ETN.

Another noticeable signal was ETN5–5, which was also detected by both *nonind* subpopulation and the whole population (HN_nonind and HN_whole) (Fig. 2b). A WUSCHEL-related homeobox (WOX) transcription factor gene *DWT1-LIKE2* (*DWL2*) was thought to be the candidate. The previous report has shown that DWARF TILLER 1 (DWT1), a WOX transcription factor, act as a positive regulator of tiller growth (Wang et al. 2014) and a DWT1 homolog DWL2 shares partial functional redundancy with DWT1 in controlling uniform growth of rice tillers and main shoot (Fang et al. 2020). *DWL2* was located 38 kb downstream of the lead SNP (rs5_28062171). LD heatmap showed a moderate LD level around the *DWL2* gene (Fig. 3d). A total of 12 SNPs were detected in the promoter region, while no non-synonymous SNP or InDel was found in CDS (Fig. 3e). Haplotype analysis revealed that Hap3 and Hap6 are favorable alleles for ETN and preferentially exist in *nonind* subgroup (Fig. 3f). Like *OsAAP1*, these two alleles of *DWL2* from *nonind* subgroup may also be useful for enhancement of ETN when introduced to *Indica* cultivars.

**Fig. 4:**
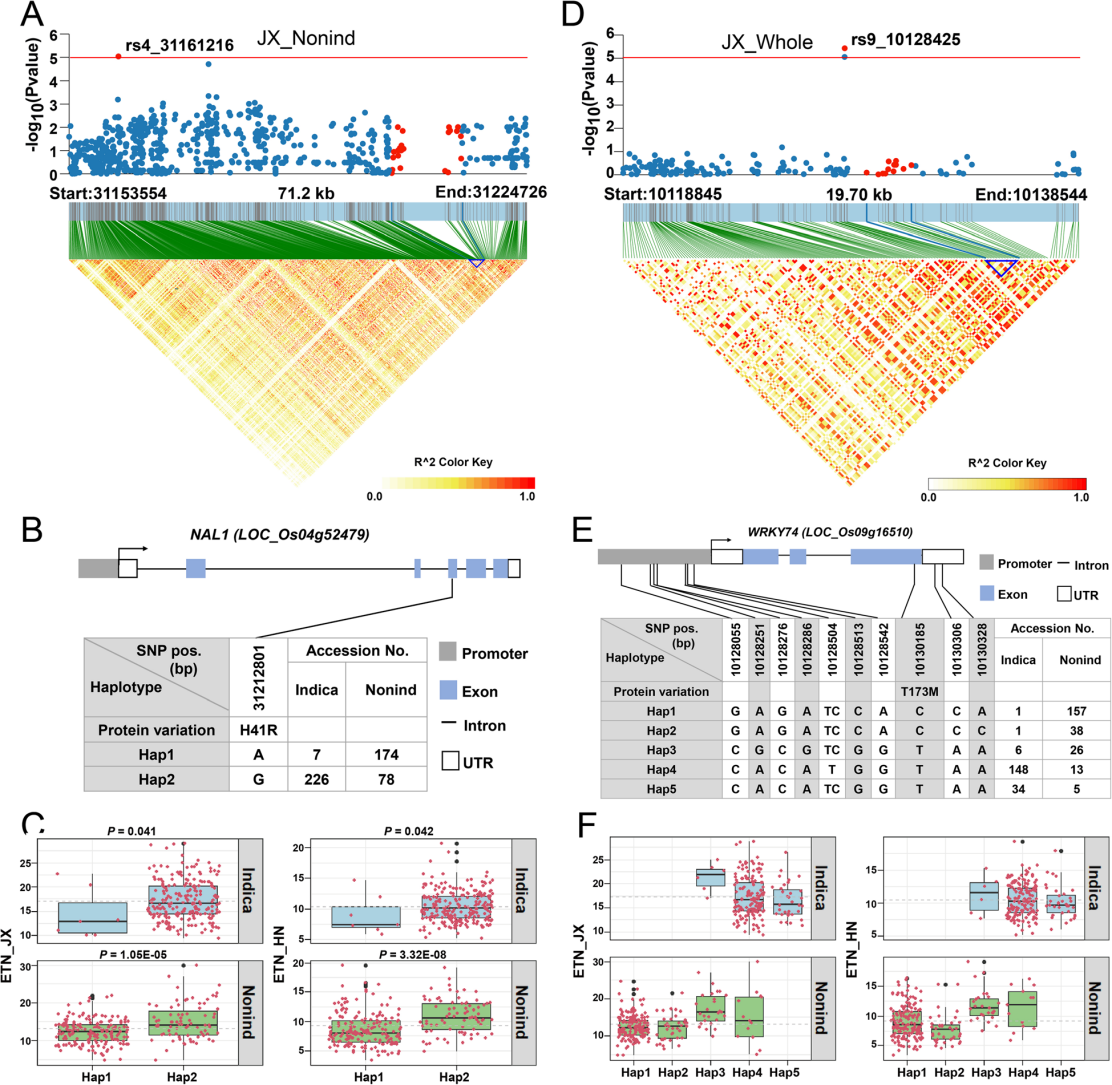
Associated region and haplotype analysis of *NAL1* and *OsWRKY74*. (**a, d**) Regional Manhattan plots and LD heatmap of *NAL1* (**a**) and *OsWRKY7* (**c**). The single red dot above the red line denotes the lead SNP, the red dots under the red line and the blue triangle in the LD heatmap denote the genomic region of candidate genes. (**b, e**) Gene structure and marker information of *NAL1* (**b**) and *OsWRKY74* (**e**)*.* Arrow heads indicate the direction of gene transcription. (**c, f**) Haplotype analysis of *OsAAP1* (**c**) and *OsWRKY74* (**f**). Boxplot described ETN distribution of one subpopulation with one haplotype, and each red dot around the boxplot represents ETN value of one accession. The gray dotted-lines denote average ETN of one subgroup. Different letters denote significant differences (*P* < 0.05) based on Duncan’s multiple-range test

Within the QTL ETN4–5 detected in JX_nonind population (Fig. 2a), we identified the *Narrow Leaf1* (*NAL1*) gene, which was initially cloned from a loss-of-function mutant showing narrow leaf and increased TN (Qi et al. 2008; Jiang et al. 2015). We identified a non-synonymous SNP that was previously reported to be associated with panicle number (Yano et al. 2016) (Fig. 4b). The results demonstrated that Hap2 with G allele showed more ETN than Hap1 with A allele in both *indica* and *nonind* subgroup (Fig. 4c). Furthermore, this SNP was also identified in a major QTL *LSCHL4* from *Japonica* cultivar, which can increase grain yield when introduced into the *Indica* super rice variety 93–11 (Zhang et al. 2014).

Within the QTL ETN9–3 detected in the JX_whole population (Fig. 2a), the *OsWRKY74* gene encoding a WRKY transcription factor was identified. *OsWRKY74* was involved in tolerance of phosphate starvation, whose overexpression led to a 24% increase in TN (Dai et al. 2015). Six SNPs and one InDel were found in the promoter region, and one non-synonymous SNP was found in CDS (Fig. 4e). There are five haplotypes in total for *OsWRKY74*, among which Hap3 is the favorable allele for *indica* subgroup, whereas Hap3 and Hap4 are favorable alleles for *nonind* subgroup (Fig. 4f). The colocalization analysis above suggested that our GWAS results were reliable because several previously reported TN-associated genes were identified in this study.

Next, we carried out an* in silico* analysis of the pyramiding effect of the favorable alleles of the above four genes on ETN. Among the *nonind* subgroup, 34, 42, 78 and 39 accessions harbor Hap5 of *OsAAP1*, Hap3/6 of *DWL2*, Hap2 of *NAL1*, Hap3/4 of *WRKY74*, respectively. Interestingly, there are 18 accessions harboring favorable alleles of all the four genes (Additional file 5: Fig. S2a), which showed more ETN compared with accessions with favorable allele of only one gene. Pyramiding favorable alleles of four genes led to 60.2% and 56.5% increase in ETN compared with the average ETN of *nonind* subgroup in Jiangxi and Hainan (Additional file 5: Fig. S2 b, c), respectively.

### Identification of Novel ETN-Associated QTLs

We aimed to identify novel ETN-associated QTLs in two ways. One is mining genes homologous to known tillering-related genes, and another is mining genes involved in plant hormone synthesis or signal transduction, including SL, auxin, cytokinin and GA. In total, we identified 25 novel candidate genes that may be related to ETN. Among them, 10 genes are involved in auxin synthesis or signal transduction, three genes participate in cytokinin synthesis, two genes participate in carotenoids synthesis, one gene participates in GA signal transduction (Yamaguchi et al. 2008), two genes encode ammonium transporter, two genes encode peptides/amino acid transporter and five genes encode sugar/glucose transporter. Detailed information of these 25 genes is listed in Additional file 2: Table S2.

**Fig. 5:**
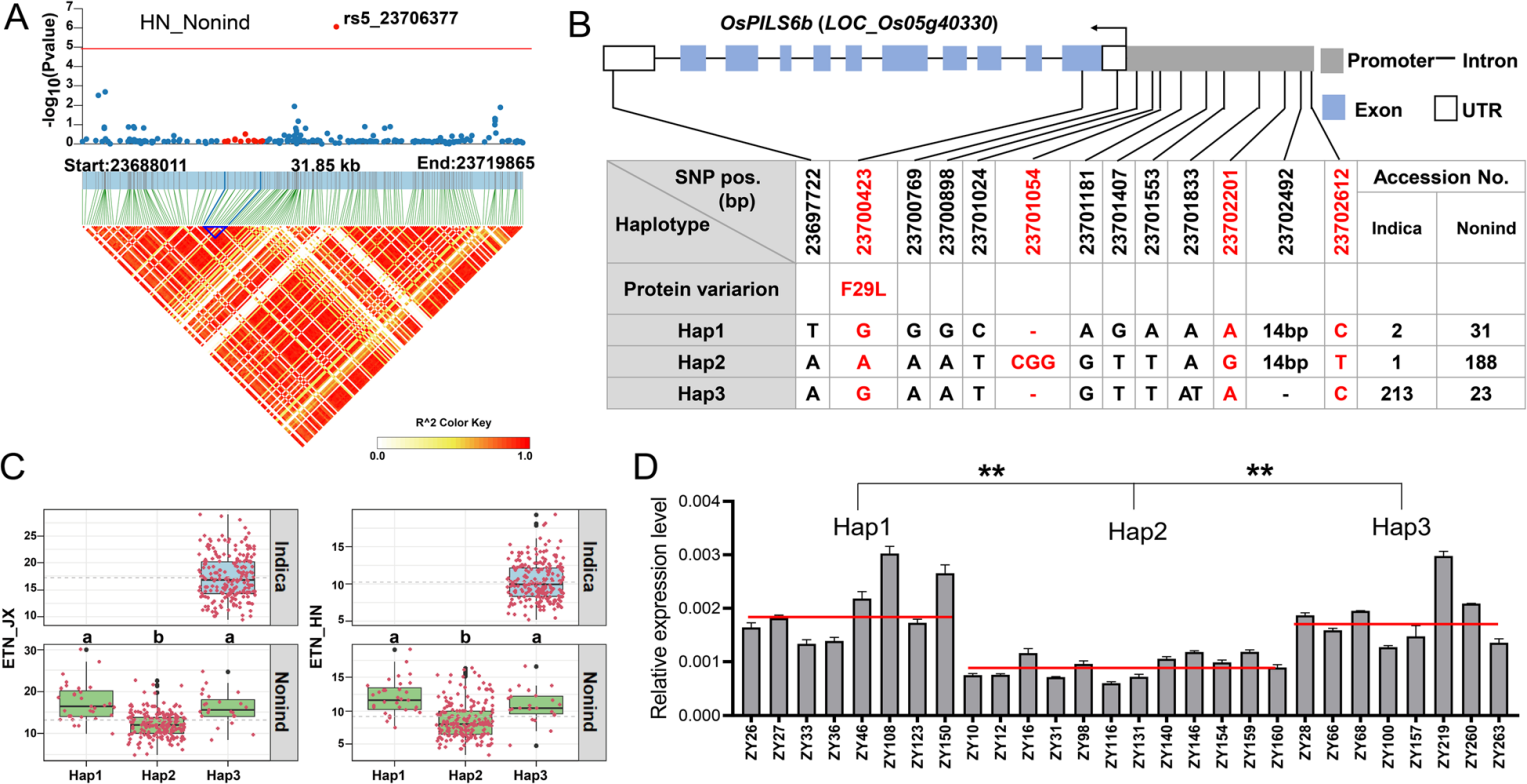
Haplotype and transcription analysis of *OsPILS6b*. (**a**) Regional Manhattan plots and LD heatmap of *OsPILS6b.* (**b**) Gene structure and SNP information of *OsPILS6b*. Red characters marked the specific variations for Hap2. The 14-bp deletion in Hap3 is AAAATGGCGGATTG. (**c**) Haplotype analysis of *OsPILS6b*. Boxplot described ETN distribution of one subpopulation with one haplotype, and each red dot around the boxplot represents ETN value of one accession. The gray dotted-lines denote average ETN of one subgroup. Different letters denote significant differences (*P* < 0.05) based on Duncan’s multiple-range test. (**d**) The relative expression level of *OsPILS6b* in plants with different haplotypes. Red lines represent the average expression level of accessions with three haplotypes, respectively. “ZYx” is the field number and the detailed information was listed in Additional file 1: Table S1. Data are mean ± SEM, ** means Student’s *t*-test, *P* < 0.01

Among the 25 novel candidate genes, we selected the *OsPILS6b* gene (*LOC_Os05g40330*) for further analysis, which encodes an auxin efflux carrier protein. Several members of this gene family have been reported to affect rice tillering, including *OsPIN2* (Chen et al. 2012), *OsPIN5b* (Lu et al. 2015a) and *OsPIN9* (Hou et al. 2021). LD analysis showed that *OsPILS6b* located in a relatively high LD region (Fig. 5a). A total of 8 sequence variations were identified in *OsPILS6b*, including five SNPs and one InDel in the promoter region, one non-synonymous SNP in CDS, and one SNP in 3′-UTR (Fig. 5b). Based on these variations, three haplotypes were identified. The *indica* subgroup has only one haplotype (Hap3) while the *nonind* subgroup has all the three haplotypes. Interestingly, most *nonind* accessions were found in Hap2 and had relatively low ETN (Fig. 5c). Considering the fact that overexpression of *OsPIN2* and *OsPIN9* have been reported to increase tiller number (Chen et al. 2012; Hou et al. 2021), we detected the *OsPILS6b* expression levels in accessions with different haplotypes. As expected, accessions with Hap2 had lower expression levels in *OsPILS6b* than those with Hap1 and Hap3 (Fig. 5d), indicating that the expression level of *OsPILS6b* is indeed associated with rice tillering.

To find out the causative variation for the lower expression in *OsPILS6b* in accessions with Hap2, we resequenced the *OsPILS6b* genomic region of the 28 accessions used for RT-qPCR, including 2.5 kb upstream of the start codon ATG. The result showed that besides the variations described above, two SNPs and one 3-bp InDel specific for Hap2 were detected in the promoter region of *OsPILS6b* (Fig. 5b). We suppose these variations affect the regulation activity of some transcription factors (TF), thus affect the *OsPILS6b* gene expression. Therefore, we extracted the 100 bp flanking sequence (50 bp upstream and 50 bp downstream) of the three variation and predicted the TF binding sites using the online tool PlantRegMap (https://plantregmap.gao-lab.org/) (Tian et al. 2020). A total of 5, 3 and 261 potential binding sites were located in the flanking sequences of two SNPs and the 3-bp InDel (Additional file 4: Table S4). Notably, it seems that the sequence near the 3-bp InDel is a binding hotspot for multiple transcription factors. Thus we speculate that this InDel is most likely the responsible site for the different expression levels of *OsPILS6b* among the three halplotypes.

### Temporal Expression Pattern of ETN-Associated Genes

**Fig. 6:**
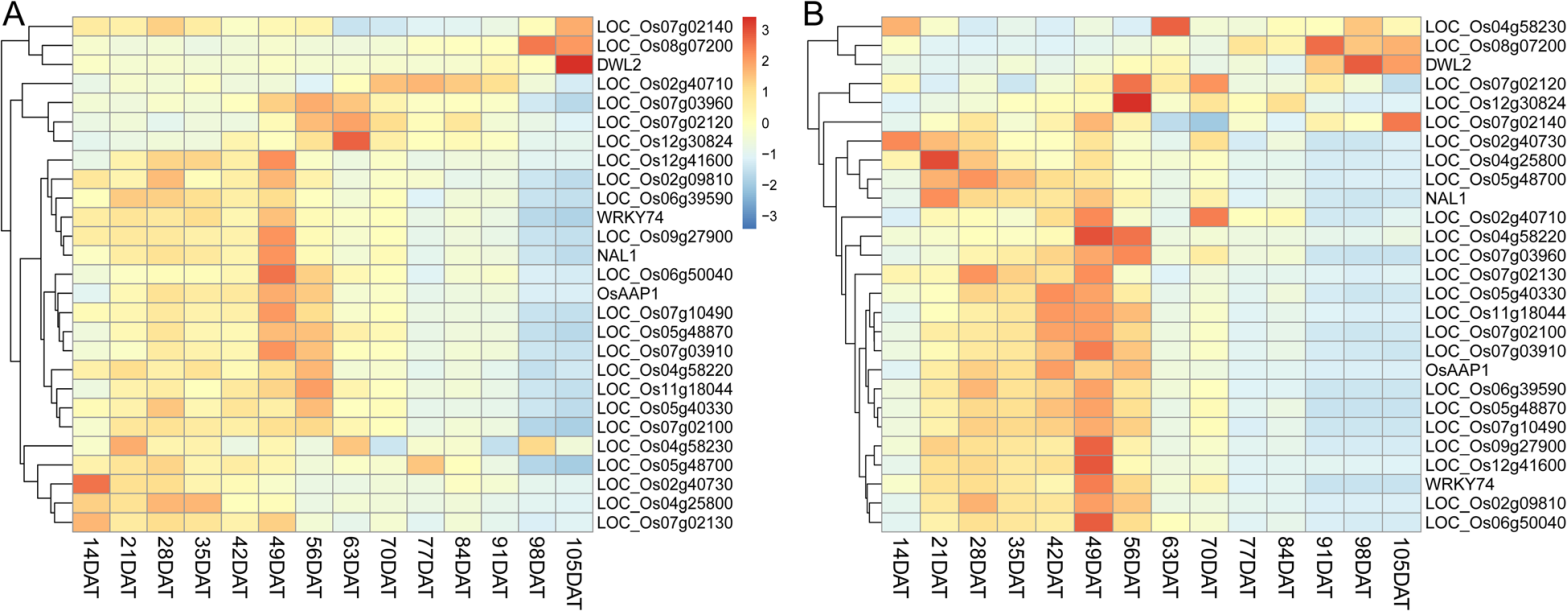
Temporal expression pattern of ETN-associated genes during the whole growth period. Expression data in root at 00:00 (**a**) and 12:00 (**b**) were downloaded from RiceXPro website (https://ricexpro.dna.affrc.go.jp/). The heatmaps represented hierarchical clustering of relative expression levels of 27 candidate genes at different days after transplanting (DAT). The scale for relative expression levels (after normalized by z-score) is denoted by color bars, with red representing the high expression levels, white medium expression and blue low expression

A recent study reported that most tillering-related genes showed a special spatio-temporal expression pattern during the whole growth period. Their expression in root at 00:00 (R0) and 12:00 (R12) are steadily high from 20 days after transplanting (DAT) to 48 DAT and then decrease after 48 DAT (Ma et al. 2020). This pattern may genetically explain the steady transition from tiller development to panicle development. Enlighted by this, we investigated the temporal expression pattern of the ETN-associated genes identified in this study. We did expression clustering analysis using the expression data of root collected at 00:00 (R0) and 12:00 (R12) with weekly interval during the whole growth period from RiceXPro website (http://ricexpro.dna.affrc.go.jp/) (Sato et al. 2011). As expected, among the 27 genes with expression data available in RiceXPro (Additional file 3: Table S3), 17 and 20 genes showed a higher expression from 21 DAT to 49 DAT in R0 and R12, respectively (Fig. 6). This expression pattern coincides with the dynamic change pattern of tiller number during the whole growth period where tiller number increases rapidly from 21 DAT and reaches the peak at 49 DAT (Liu et al. 2018; Liu et al. 2021). The decreased expression of ETN-associated genes after 49 DAT may contribute to the transition from vegetative to reproductive growth of tillers and panicle development on tillers.

## Discussion

### GWAS Is a Feasible Way to Mine ETN-Associated QTL

ETN directly determines rice grain yield. Most of the previous studies on ETN were based on classical QTL mapping using biparental populations (Xu and Shen 1991; Wu et al. 1999; Yang et al. 2006; Liu et al. 2010), and few of these QTLs were cloned actually. Traditional QTL mapping can only exploit genetic variations in two parents. However, GWAS can take advantage of extensive variations in numerous natural population (Wang et al. 2020b). Using a panel of 490 rice accessions selected from the 3000 rice genomes project (Wang et al. 2018) and millions of SNPs derived from the same project, we performed GWAS of ETN in two locations. We identified 38 ETN-associated QTLs and found that 1134 genes located in these QTL regions. A recent study on transcriptional regulation of strigolactone signaling in Arabidopsis revealed that genes involved in microtubule function were up-regulated whereas auxin-inducible genes were down-regulated after GR24 treatment for 2 h (Wang et al. 2020a). Among the 1134 genes identified from our GWAS results, three genes are involved in microtubule function. *LOC_Os04g58130* (*OsKTN80b*) encodes a katanin protein showing microtubule-severing activity, *LOC_Os05g02670* encodes a kinesin protein involved in microtubule-based movement, and *LOC_Os09g27700* encodes a microtubule-associated protein (MAP65/ASE1). Besides, 10 genes associated with auxin synthesis or signal transduction pathways were detected (Additional file 2: Table S2), consistent with the fact that plant hormone synthesis or signal transduction have a significant impact on rice tillering. These results hinted that genes detected from our GWAS results potentially play a role in rice tillering regulation.

Some other identified genes are also involved in plant hormones or homologous to known tillering-related genes. For instance, *LOC_Os06g50040* (*OsSAUR29*) and *LOC_Os12g41600* (*OsSAUR57*), two small auxin-up RNA genes, are homologues of *SAUR39* and *SAUR27* which have a negative effect on rice tillering (Kant et al. 2009; Ma et al. 2020). *LOC_Os07g10490* (*ZDS2*) encodes a zeta-carotene desaturase involved in the biosynthesis of carotenoids, the precursor of the plant hormone strigolactone. Indeed, genes involved in carotenoids biosynthesis have been reported to regulate rice tillering via the carotenoid-dependent strigolactone biosynthesis, such as the *MIT1* gene encoding 15-cis-ζ-carotene isomerase (Z-ISO) (Liu et al. 2020; Zhou et al. 2021; Liu et al. 2021) and the *MIT3* gene encoding carotenoid isomerase (CRTISO) (Liu et al. 2018). *LOC_Os02g09810* encodes an amino acid transporter-like protein, and four members of this gene family i.e., *OsAAP1* (Ji et al. 2020), *OsAAP3* (Lu et al. 2018), *OsAAP4* (Fang et al. 2021) and *OsAAP5* (Wang et al. 2019) have been known to affect tillering.

### ETN-Associated QTLs Have Potential for Molecular Breeding

We detected four genes previously reported to be associated with ETN, i.e., *OsAAP1*, *DWL2*, *NAL1,* and *OsWRKY74*. Haplotype analysis revealed that these genes showed different haplotype patterns in the two subpopulations and different ETN variation among haplotypes. Among the four genes, a favorable allele of *NAL1* from *japonica* has already been used to improve yield in modern *indica* cultivars (Zhang et al. 2014; Fujita et al. 2013). As to the other three genes, there is no report about their breeding value yet. However, our results showed that Hap5 of *OsAAP1*, Hap3 and Hap6 of *DWL2*, Hap3 and Hap4 of *WRKY74* are favorable alleles for ETN (Fig. 3 and Fig. 4), and the 18 accessions harboring favorable alleles of all these four genes showed an apparent increase in ETN (Additional file 5: Fig. S2), indicating that pyramiding multiple favorable alleles may contribute to high ETN.

Besides the four known genes, we identified 25 novel genes that may also affect ETN, and the *OsPILS6b* gene encoding a PIN family member was selected for preliminary validation through RT-qPCR. Accessions with Hap1 and Hap3 generally have higher ETN than accessions with Hap2. Interestingly, the expression levels of *OsPILS6b* were also higher in accessions with Hap1 and Hap3 than those with Hap2 (Fig. 5). This result is consistent with previous findings that *OsPIN2* and *OsPIN9,* two homologs of *OsPILS6b*, contribute positively to tillering (Chen et al. 2012; Hou et al. 2021). Moreover, using the public expression data from RiceXpro website, we identified a common spatio-temporal expression pattern for ETN-associated genes, which showed a relatively higher expression from 21 DAT to 49 DAT in root and decreased after that (Fig. 6). This expression pattern coincides with the dynamic change pattern of tiller number during the whole growth period (Liu et al. 2018; Liu et al. 2021). We also analyzed the haplotypes of some other genes, including *LOC_Os07g10490* (*ZDS2*), *LOC_Os06g39590* (*OsIAA23*), *LOC_Os06g50040* (*OsSAUR2*9) and *LOC_Os12g41600* (*OsSAUR57*) (Additional file 5: Fig. S3). A total of 5, 9, 11, 8 haplotypes were identified for these four genes, respectively. In addition, these four genes displayed different haplotype patterns between the *indica* and *nonind* subpopulation, indicating that some of the ETN-associated genes differentiated after the differentiation of *japonica* and *indica* subspecies. These results enlighten us to further mine the favorable alleles in natural resources which can be used for in rice breeding for high ETN.

## Conclusions

In this study, we identified 38 ETN-associated QTLs through GWAS using a panel of 490 rice accessions. Four QTLs were colocalized with known genes involved in tillering regulation, and 25 novel genes located in these QTL regions were related to plant hormones or homologous to other ETN-associated genes. The favorable alleles mined with GWAS have potential value in rice molecular breeding for high ETN.

## Supplementary Information

**Additional file 1: Table S1.** Summary of the 490 rice accessions and their ETN phenotypes and information of 28 accessions used for *OsPILS6b* transcription analysis.

**Additional file 2: Table S2.** Detailed information of significant SNPs and ETN-associated genes mapped by six GWAS assays.

**Additional file 3: Table S3.** Expression data of 27 ETN-associated genes.

**Additional file 4: Table S4**. Potential transcription factors and their binding sites in the selected regions of *OsPILS6b* promoter.

**Additional file 5: Figure S1**. Cross-validation plot for population structure and linkage disequilibrium analysis. **Figure S2.** *In silico* analysis of the pyramiding effect of favorable alleles of *OsAAP1*, *DWL2*, *NAL1* and *WRKY74*. **Figure S3.** Haplotype analysis of *ZDS2*, *OsIAA23*, *OsSAUR2*9 and *OsSAUR57*.

## Data Availability

All data generated or analyzed in this study are available within the manuscript or its supplementary files or are available from the corresponding authors upon request.

## Abbreviations

3 K RGP: 3000 Rice Genomes Project
ANOVA: Analysis of variance
AM: Axillary meristem
CDS: Coding sequence
EMMAX: Efficient mixed model association expedited
ETN: Effective tiller number
GAs: Gibberellins
GWAS: Genome-wide association study
HN: Hainan Province
InDel: Insertion and deletion
JX: Jiangxi Province
LD: Linkage disequilibrium
MAF: Minor allele frequency
NGS: Next-generation sequencing
PCA: Principal component analysis
QTL: Quantitative trait locus
RT-qPCR: Reverse transcription quantitative-PCR
SNP: Single nucleotide polymorphism
SLs: Strigolactones
TN: Tiller number
UTR: Untranslated region
