# Supplementary material for: Genome-Wide Association Study of the Genetic Basis of Effective Tiller Number in Rice
Source: Rice (N Y). 2021 Jun 25;14:56. doi: 10.1186/s12284-021-00495-8 (PMC8233439; doi:10.1186/s12284-021-00495-8)
Supplement: Supplementary file 5 — Additional file 5: Figure S1. Cross-validation plot for population structure and linkage disequilibrium analysis. Figure S2. In silico analysis of the pyramiding effect of favorable alleles of OsAAP1, DWL2, NAL1 and WRKY74. Figure S3. Haplotype analysis of ZDS2, OsIAA23, OsSAUR29 and OsSAUR57. [file 12284_2021_495_MOESM5_ESM.docx]

**Genome-wide Association Study of the Genetic Basis of Effective Tiller Number in Rice**

Mengmeng Ren , Minghan Huang , Haiyang Qiu , Yan Chun , Lu Li , Ashmit Kumar , Jingjing Fang, Jinfeng Zhao, Hang He, Xueyong Li


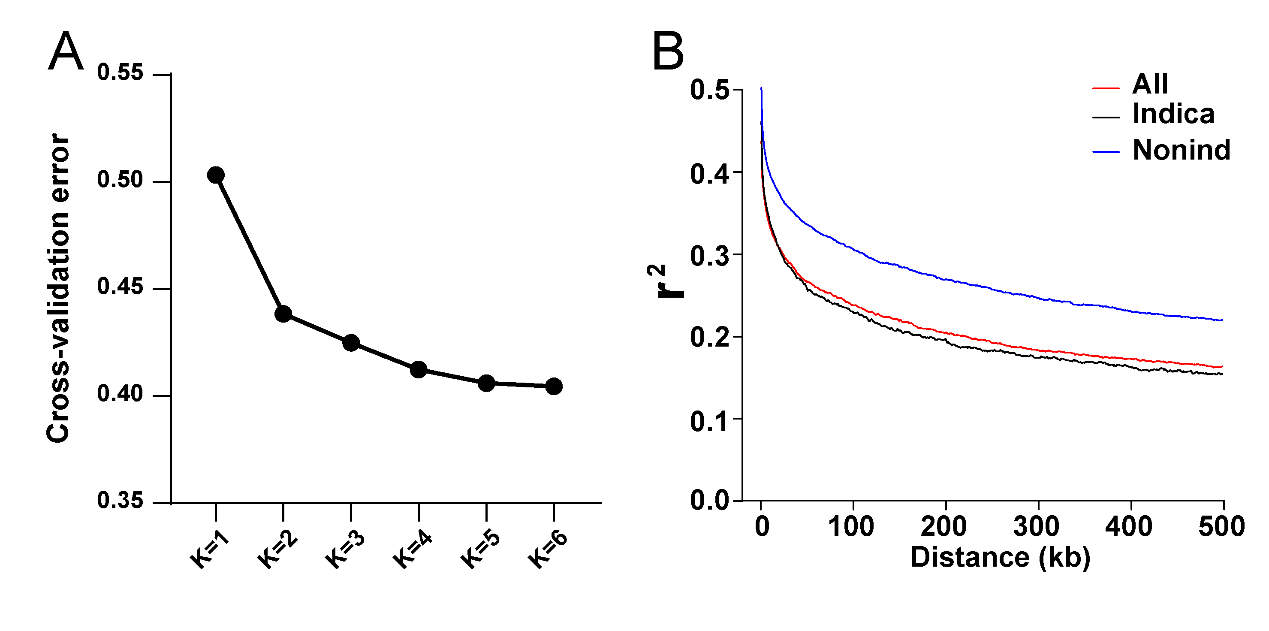


**Fig. S1**. Cross-validation plot for population structure and linkage disequilibrium analysis.(a) Five-fold cross-validation was performed using ADMIXTURE 1.3 Software. The number of ancestral populations (K-value) was assumed ranging from 2 to 6. (b) Genome-wide LD decay for the whole, *indica* and *nonind* population. The distance of LD decay (*r*2 dropped to half of the maximum value) for the whole, *indica* and *nonind* population is 128, 112 and 296 kb, respectively.


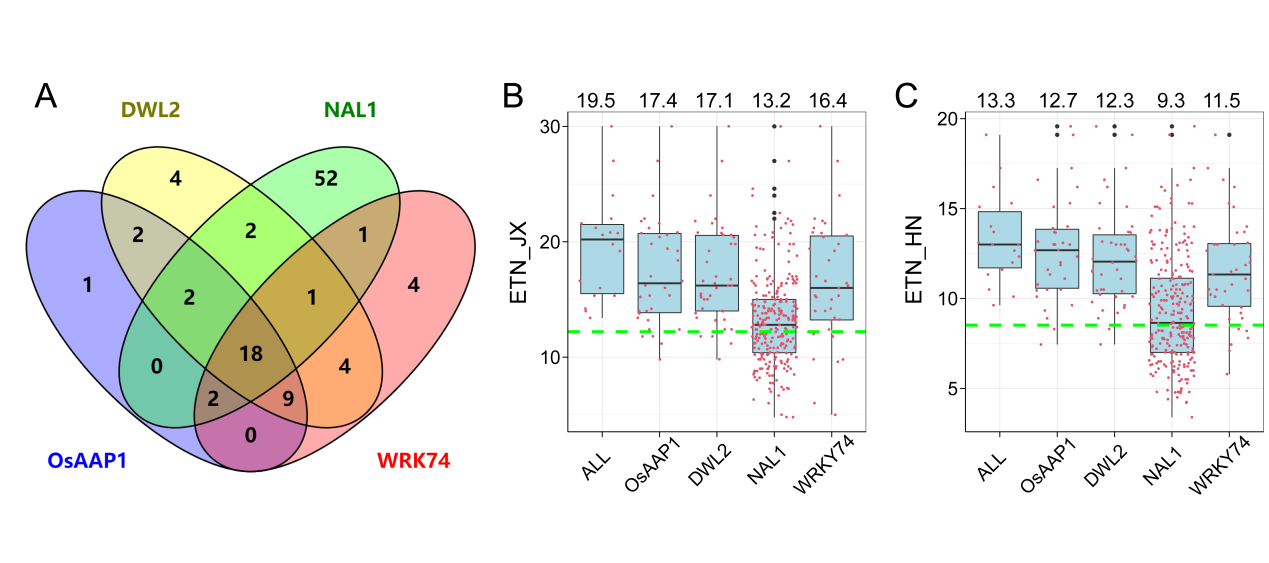


**Fig. S2** *In silico* analysis of the pyramiding effect of favorable alleles of *OsAAP1*, *DWL2*, *NAL1* and *WRKY74*. (**a**) Venn diagram of accessions harboring favorable alleles (Hap5 of *OsAAP1*, Hap3/6 of *DWL2* , Hap2 of *NAL1*, Hap3/4 of *WRKY74*) in *japonica* subgroup. (**b, c**) Boxplot of ETN of accessions harboring favorable alleles of four genes (ALL) and accessions harboring favorable allele(s) of only one gene (*OsAAP1*, *DWL2*, *NAL1* and *WRKY74*) in Jiangxi (**b**) and Hainan (**c**). Each red dot around the boxplot represents ETN value of one accession. The number above each boxplot represents the average ETN of each group. The green dotted line denotes average ETN of *japonica* subgroup.


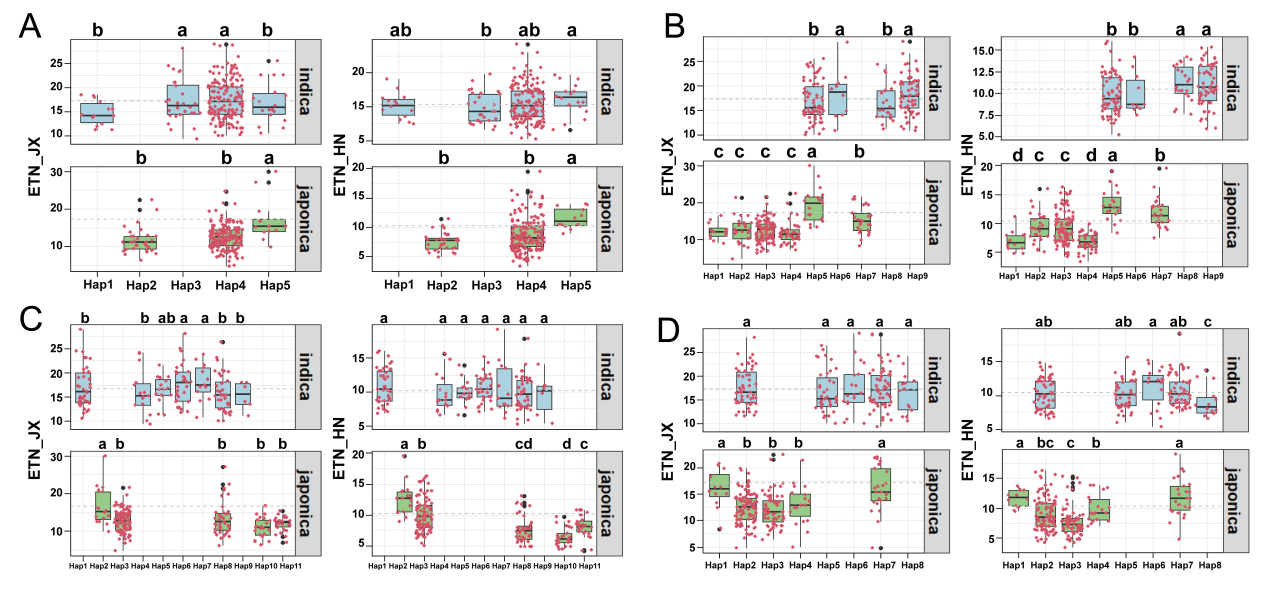


**Fig. S3** Haplotype analysis of *ZDS2* (**a**), *OsIAA23* (**b**), *OsSAUR2*9 (**c**) and *OsSAUR57* (**d**). Each red dot around the boxplot represents ETN value of one accession. The grey dotted-lines denote average ETN of one subgroup. Different letters denote significant differences (*P* < 0.05) based on Duncan's multiple-range test.
